# Yet Another Empty Forest: Considering the Conservation Value of a Recently Established Tropical Nature Reserve

**DOI:** 10.1371/journal.pone.0117920

**Published:** 2015-02-10

**Authors:** Rachakonda Sreekar, Kai Zhang, Jianchu Xu, Rhett D. Harrison

**Affiliations:** 1 Key Laboratory of Tropical Forest Ecology, Xishuangbanna Tropical Botanical Garden, Chinese Academy of Sciences, Menglun, China; 2 Ecology, Conservation, and Environment Center (ECEC), State Key Laboratory of Genetic Resources and Evolution, Kunming Institute of Zoology, Chinese Academy of Sciences, Kunming, China; 3 University of the Chinese Academy of Sciences, Beijing, China; 4 Key Laboratory for Biodiversity and Biogeography, Kunming Institute of Botany, Chinese Academy of Sciences, Kunming, China; 5 World Agroforestry Centre, East Asia Node, Heilongtan, Kunming, Yunnan, China; University of Brasilia, BRAZIL

## Abstract

The primary approach used to conserve tropical biodiversity is in the establishment of protected areas. However, many tropical nature reserves are performing poorly and interventions in the broader landscape may be essential for conserving biodiversity both within reserves and at large. Between October 2010 and 2012, we conducted bird surveys in and around a recently established nature reserve in Xishuangbanna, China. We constructed a checklist of observed species, previously recorded species, and species inferred to have occurred in the area from their distributions and habitat requirements. In addition, we assessed variation in community composition and habitat specificity at a landscape-scale. Despite the fact that the landscape supports a large area of natural forest habitat (~50,000 ha), we estimate that >40% of the bird fauna has been extirpated and abundant evidence suggests hunting is the primary cause. A large proportion (52%) of the bigger birds (>20 cm) were extirpated and for large birds there was a U-shaped relationship between habitat breadth and extirpation probability. Habitat specificity was low and bird communities were dominated by widespread species of limited conservation concern. We question whether extending tropical protected area networks will deliver desired conservation gains, unless much greater effort is channeled into addressing the hunting problem both within existing protected areas and in the broader landscape.

## Introduction

Habitat degradation and loss are commonly regarded as the most important threats to biodiversity globally, driving local extinctions and skewing the abundances of persisting species [1,2]. Human driven habitat degradation has intensified during the past 50 years and is likely to accelerate during the next 50 years [3]. In the last decade (2000–2010) alone, around 130 million hectares of forests were lost [4]. In the face of this loss and degradation of natural habitat, one of the most common measures for conserving biodiversity is in the use of protected areas, which ideally act as a repository of native biodiversity and natural ecosystem processes [5]. However, it is essential that assessments of the effectiveness of this approach are made [6–8]. A now substantial literature has examined the extent of protected area coverage (e.g., [5,9–12]) and several studies have investigated the impacts of reserves on deforestation rates or habitat loss (e.g., [5,13,14]). However, these are at best proxy measures of conservation gains. Relatively few studies have assessed how effective tropical protected areas have been in protecting the biodiversity they were set up to conserve, especially in the tropics (but see [5,15,16]). Most studies do not assess plant and animal populations directly, but have focused on easily assessed indicators of success that may be poor measures of biodiversity, especially at local scales [9]. Moreover, in the past the effectiveness of reserves was often exaggerated (e.g., [16]), because of a failure to distinguish between the effects of the intervention and the geographical circumstances that may have preserved biodiversity historically. This is critical because development in areas surrounding reserves often introduces new threats to biodiversity [5,17–19].

Management of biodiversity in the broader landscape is often of critical importance for the maintenance of biodiversity [20–22] and the quality of enforcement in tropical protected areas is highly variable [5]. In particular, hunting is an increasingly serious problem for the conservation of many species both within protected areas and in the broader landscape [23–26]. ‘Empty forests’ or ‘defaunated forests’ are somewhat evocative terms that have been used by researchers to denote forests with drastically depleted vertebrate populations [27–29]. The terms convey the notion of a forest that remains structurally and botanically intact (or at least that is not heavily degraded), but whose larger and more charismatic animals have been hunted out. Throughout the tropics there are large swathes of good forest habitat that are devoid of animals larger than approximately 1 kg, barring a few hunting tolerant species [23,25].

In tropical Asia, pressure on biodiversity is particularly severe. Historically high human population sizes have resulted in a low proportion of remaining forest cover, and industrial plantations, poverty and poorly regulated land developments have combined to produce high rates of deforestation [30]. Large wilderness areas are absent over much of the region and hence protected areas are often established within a matrix of smaller natural forests, agricultural landscapes, plantations and human settlements [21]. In addition, these complex landscapes are often inhabited by diverse ethnic groups, whose livelihoods include collection and use of wild natural resources [20,31]. Management of such areas thus is complicated in both ecological and social dimensions.

Yunnan Province, China, comprises a transition zone from tropical SE Asia to subtropical East Asia, making it one of the globe’s most biologically diverse regions [32]. Xishuangbanna is Yunnan’s southernmost prefecture and the most tropical in character. In recent years, a large proportion of the natural forest in Xishuangbanna has been converted to monoculture plantations, especially rubber and tea [8]. However, nature reserves cover >18% of the prefecture and a comprehensive system of biological corridors has been proposed [33]. As with elsewhere in SE Asia, most of these reserves have significant numbers of indigenous people living within or near their boundaries [34,35]. Thus, in terms of the difficulties facing biodiversity conservation, in many respects Xishuangbanna represents a microcosm of tropical SE Asia. Using birds, we evaluate the contribution a recently established protected area makes to biodiversity conservation in Xishuangbanna. We focused on this area because of the availability of comprehensive baseline data on birds, forest structure and socio-economics [35–37].

Specifically, we addressed the following questions. 1) How does bird species richness and composition vary across the landscape, both within the reserve and its immediate environments? 2) Which species occur within the landscape today and which species are known or inferred to have occurred there in the past? And 3) what traits best explain extirpation risk? We found that, despite having a relatively large area of natural forest habitat, the bird fauna was depauperate and that intense local hunting is the most likely cause of extirpations. We discuss the implications of our findings for the conservation of tropical biodiversity.

## Materials and Methods

### Study area

**Fig 1:**
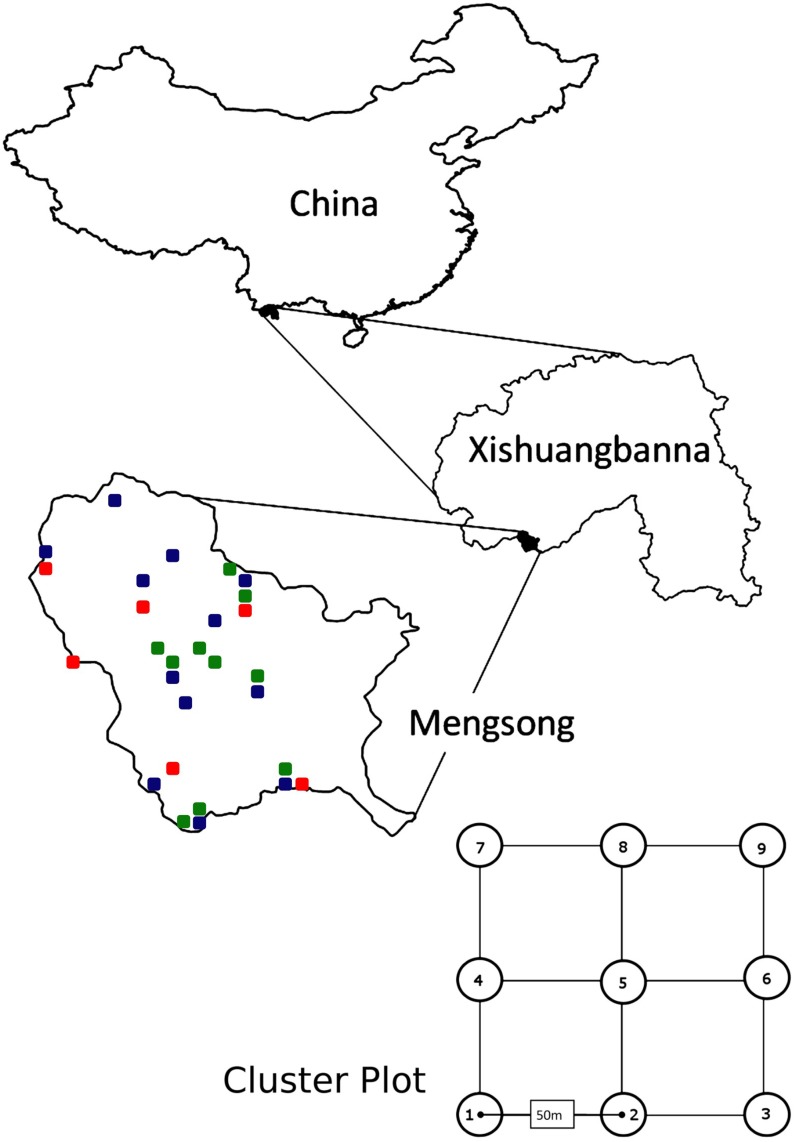
Map of the study site in Xishuangbanna Prefecture, China, and 28 plots across a disturbance gradient (green: near-pristine forest; blue: degraded forest; red: open landscape) in the Mengsong Township. Each plot comprised nine subplots in a 3 × 3 array with 50 m spacing between subplots.

We conducted this study in Mengsong district (Xishuangbanna prefecture, Yunnan, China; Fig. 1), a landscape of approximately 100 km^2^ with multiple villages and hamlets that borders Myanmar and is an important sub-watershed of the Mekong River. The elevation varies from 800 m to 1800 m and steep valleys dissect the area. Mengsong has a tropical seasonal climate influenced by the Indian monsoon with an annual mean temperature of 18°C (at 1600 m asl) and an annual rainfall between 1600–1800 mm [35]. Evergreen tropical seasonal rain forests dominate in the valleys and tropical montane forests dominate ridges, while subtropical evergreen broadleaf forest forms a transitional zone on slopes [35]. In 2009, the Bulong Nature Reserve was established in Mengsong and the neighboring district of Bulong as a part of a conservation program that aims to connect all the remaining native forest in Xishuangbanna [33]. The reserve encompasses 36,000 ha, with additional forest in the landscape outside the reserve bringing the total forest area up to approximately 50,000 ha. Most of this forest is secondary re-growth or degraded primary forest, but approximately 25% is botanically near-pristine.

The population settled in Mengsong is dominated by Akha people (referred to as Hani in Chinese publications), a local ethnic group who practised swidden cultivation for around 200 years in the region until logging was banned in 1998 [35]. The Akha have subsequently increased cultivation of cash crops, such as tea.

### Bird surveys

We conducted a landscape-scale survey of the birds of Mengsong to fulfill two purposes. First, we wished to assess the level of differentiation among bird assemblages across a disturbance gradient from botanically near-pristine forests to open-land (see section ‘*Bird assemblages across a disturbance gradient*’). Second, we wished to ensure that the entire landscape was well sampled to produce a reliable checklist of extant birds (see section ‘*Extirpated birds*’). Thus, our sampling involved high replication at a landscape-level rather than intense sampling of a smaller area.

### Bird assemblages across a disturbance gradient

#### Plot selection and habitat characteristics

We sampled 28 previously established permanent plots (R.D.H., *unpublished data*) stratified across three disturbance categories: near-pristine forest, secondary or heavily degraded forest, and open areas (terrace tea plantations and grasslands). Disturbance categories were assigned using remote sensing images, combined with ground-truthing, and plot selection was based on a stratified (by location and disturbance category) random approach, resulting in an unbiased plot selection with plots of each disturbance category interspersed across the landscape (Fig. 1). Each plot comprised nine subplots in a 3 × 3 array with 50 m spacing between subplots (Fig. 1). To characterize the habitat we measured the following characteristics in all the nine subplots and used the plot mean of each parameter (S1 Table): 1) distance to open habitat (zero for open-area plots), 2) leaf area index using a hemispherical lens and Gap Light Analyzer software [38], 3) elevation using a barometric altimeter, and 4) diameter at breast height (1.3 m; DBH) of all trees (>10cm DBH) within a 10 m radius, from which basal area was estimated.

#### Bird sampling

We sampled all 28 plots two times for birds, once in the morning and once in the evening, between October and December 2010. We surveyed five subplots per plot, selecting the subplots in the corners and the middle (as in the number five on a die), and sampled birds using a fixed-width point count. Each count was taken by waiting five minutes after walking to the subplot and then recording for 12 minutes. All bird observations (sighting and aural detections) within 30 m of the center of the subplot being surveyed were recorded. All over-flying birds and uncertain identifications were removed from the analysis. Bird calls were also recorded in the field and cross-checked at the field-station. Birds were identified by reference to MacKinnon and Phillipps [39].

#### Species richness

We used Chao’s non-parametric estimator to project the species richness in three habitat types [40]. We investigated the factors determining species richness of 1) all species (ALL), 2) widely distributed species (WD; range > 3,500,000 km^2^), 3) species with medium-sized distributions (MD; range < 3,500,000 km^2^ and > 2,300,000 km^2^), and 4) restricted range species (RD; range < 2,300,000 km^2^) [39,41]. Species with medium-sized and restricted distributions were later lumped, because there were only eight species with restricted range distributions. The estimated species richness was log-transformed before analysis to meet the assumptions of normality and homoscedasticity. The model parameters were considered significant at *P* < 0.05 and significance levels were adjusted using a Bonferroni correction for multiple comparisons.

#### Habitat specificity

We used a habitat specificity index following the approach of Tylianakis et al. [42]. The expected number of individuals of species *i* for plot *j* was calculated as *E*_*ij*_ = *N*_*i*_ × *P*_*j*_, where *N*_*i*_ is the total number of individuals of species *i* across all habitat types, and *P*_*j*_ is the proportion of all individuals across all species and habitats that were found at plot *j*. We then calculated the measure of specificity using log_10_([*O*_*ij*_ / *E*_*ij*_] + 1), where *O*_*ij*_ is the observed number of individuals of species *i* at plot *j*. Species with less than four detections were removed from the analysis to reduce the influence of accidental occurrences [43]. The specificity measures for species were then compared across habitats using a non-parametric Kruskal-Wallis test.

#### Community composition

Variance in bird species composition across the landscape was assessed with both abundance (weighted) and presence-absence (unweighted) data. However, we only present results from the abundance weighted analysis, as there was no qualitative difference in results between these analyses. We used a multivariate generalised linear model (MGLM) with negative binomial errors for abundance data and a MGLM with binomial errors for presence-absence data. We used distance to open habitat, leaf area index and elevation as predictor variables (function *manyglm* in the package *mvabund* in R v2.15 [44]). Basal area was not included in the model, because it was highly correlated with leaf area index (r = 0.90, *P <* 0.001). Species with less than four detections were removed from the abundance data prior to analysis. However, all the species were retained in the presence-absence data. The estimated *P*-values were obtained from monte-carlo resampling (999 random permutations). We used non-metric multidimensional scaling (NMDS) to visualize the bird composition data.

### Estimating bird extirpations

#### Bird survey

In addition to the plot surveys described above, we constructed checklists of both extant and extirpated birds in the Mengsong landscape. Bulong Nature Reserve was first surveyed for birds between 1998 and 2000 [36]. This survey yielded 146 species in 120 days, of which 98 were resident diurnal species. We resurveyed the study area between October 2010 and October 2012 for ~200 days (October-December 2010, 10–15 October 2011, November 2011—January 2012, July 2012, and September 2012). We surveyed the whole study area repeatedly by slowly walking through the landscape and compiled lists of all birds seen or heard till the species accumulation curve flattened. Migrants and nocturnal species were excluded from analyses, because migrants may be affected by factors acting outside the area and nocturnal species may have been overlooked. We constructed an inferred bird species checklist of species that ought to occur in the area based on range, elevation and habitat data by referring to MacKinnon and Phillipps [39], Cheng and Cheng [45,46], Ivanov [47], Yang [48], Yang et al. [49], Yang and Yang [50]. Although it is possible that such an inferred checklist could over-estimate the number of birds that were originally present in the landscape, such errors are likely to be few as all birds included in the inferred checklist were recorded within a 50 km radius of the study site [45–50]. Moreover, the approach is justified given the high rate of extirpations that must have occurred before bird surveys were conducted.

#### Bird functional traits

To investigate the correlates of extirpation among bird species, we measured nine life history and ecological traits using data from MacKinnon and Phillipps [39], Yang et al. [49], Yang and Yang [50] and Robson [51] (S2 Table). The nine traits were forest specialization (specialist or non-specialist; birds that primarily reply on natural forest habitats according to Robson [51] were classified as specialists); disturbed habitat use (occurs in disturbed habitats, e.g., secondary growth; yes or no); size (average body size); habitat breadth (observed number of habitats a species occurs in; range 1–10); diet type of a species (primary feeding guild: insectivore; vertebrate carnivore; frugivore, nectarivore and granivore); diet breadth (observed number of major diet type a species has; range 1–7); nest substrate (ground or aboveground); minimum clutch size (minimum number of eggs laid; range 1–6); and range (restricted, medium and wide distributions; see ‘species richness’ column for details) (S2 Table). Classification of bird functional traits followed the global bird ecology database (see Sekercioglu et al. [52] for details).

#### Extirpation risk

We used a classification tree procedure to evaluate the key correlates of extirpation risk in a hierarchical manner. We used a dichotomous response variable: extirpated or extant. Species that were not sighted during our study but were present in the complete checklist (S2 Table; inferred and previously recorded) were considered ‘extirpated’. The nine life history and ecological traits were used as predictor variables. These were used to grow an overlarge tree with a minimum splitting group of size one and cost complexity measure of 0.0001. Lower branches were pruned by 10-fold cross validations to produce an optimal tree, hence reducing data over-fitting, which was within 1 SE of the minimum-error tree [53]. Generalized linear models (GLM) with binomial errors and logit links were used to model the effect of the predictor variables generated by the optimal tree on the extirpation probability of birds. We used the *rpart* package in R v2.15 to build the classification tree model [44].

### Hunting pressure

As an index of hunting pressure, we counted the number of people carrying shotguns on trails, while walking to and from plots. People in Mengsong often hunt birds during the day and do not conceal their activities. However, as much hunting activity also takes place at night, our measure can only be regarded as a crude index of total hunting pressure.

Twenty (seven near-pristine, nine degraded and four open habitats) among the 28 plots were sampled five times over a year. The number of hunters sighted on trails was standardized by estimating the number of hunters sighted per 30 minutes and then compared across habitats using a non-parametric Kruskal Wallis test.

### Ethics statement

As this was a study based entirely on observational data, no collecting or export permits were required.

Bulong Nature Reserve is a provincial level reserve. Permission to work in the reserve was obtained under a cooperative agreement between Xishuangbanna Tropical Botanical Garden, Chinese Academy of Sciences and Xishaungbanna Nature Reserve Bureau of the Xishuangbanna Provincial Government.

## Results

### Bird assemblage across a disturbance gradient

**Fig 2:**
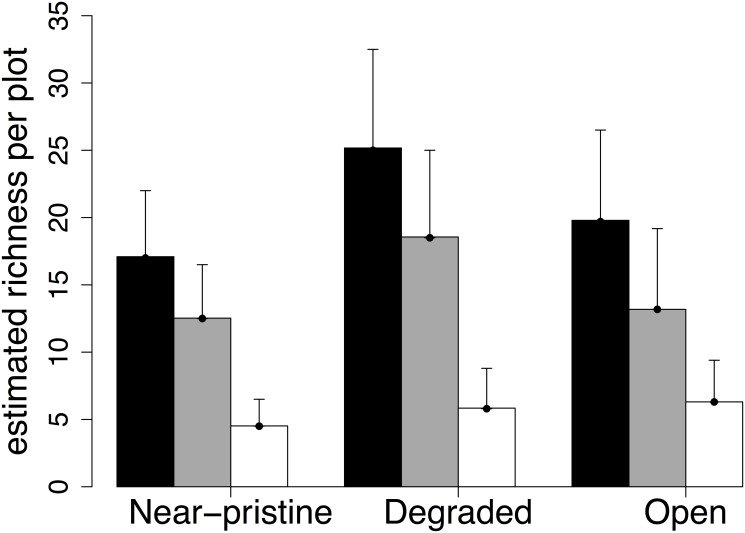
Estimated (Chao) bird species richness per plot by habitat type. Bar heights show mean estimated richness per plot and error bars represent standard deviation. Black bars represent all species, grey bars represent species with wide distributions (WD), and white bars represent species with medium and restricted distributions (MD and RD).

Of a total of 148 resident bird species recorded from Mengsong during 2010–2012 (Table 1), 83 species (55.7%, 5773 individuals) were recorded in the plots (S3 Table). Fifty-four species (65.1%) had wide distributions (WD), 21 (25.3%) had medium-sized distributions (MD) and eight (9.7%) had restricted distributions (RD). Twenty-six species (31.3%) were observed on less than four occasions. Degraded forests had higher species richness than primary forests for all species combined and species with wide distribution range, but not for medium to restricted range species (Table 2; Fig. 2). However, there was no difference in species richness between degraded forests and open landscapes or between primary forests and open landscapes (Table 2; Fig. 2).

**Table 1:** Numbers of resident bird species in Mengsong, including species occurring there in 2011–2012, in 1998–2000 (Wang and Young 2003)^a^, and inferred^b^.

	Total resident species	Wide-range^c^	Medium-range^c^	Restricted-range^c^	IUCN^d^
2010–2012	148	102 (68.9)	32 (21.6)	14 (9.4)	1
1998–2000	185	129 (69.7)	38 (20.5)	18(9.7)	1
Inferred	254	171 (67.3)	51 (20.1)	32 (12.6)	8

^a^The survey in 1998–2000 only yielded 98 resident species. A further 87 species were added by us and assumed to be present during the earlier period.

^b^Inferred is a checklist of birds that ought to occur in the study area based on range, elevation and habitat data by referring to MacKinnon and Phillipps [31], Cheng and Cheng [36, 37], Ivanov [38], Yang [39], Yang et al. [40], Yang and Yang [41].

^c^The numbers in parentheses refer to the percentage of species.

^d^IUCN Red Book Status: Near Threatened or Vulnerable or Endangered or Critically Endangered.

**Table 2:** Differences in species richness across disturbance gradients. Multiple comparisons were Bonferroni corrected, and only significant multiple comparisons are shown.

Species richness	F_2, 25_	P	Multiple comparisions	t	P
All species	3.97	0.03	Near-pristine vs Degraded	2.96	0.02
			Degraded vs Open	1.51	0.48
			Near-pristine vs Open	0.84	1
Wide distributions	3.51	0.04	Near-pristine vs Degraded	2.68	0.04
			Degraded vs Open	1.75	0.33
			Near-pristine vs Open	0.24	1
Medium to restricted distributions	0.45	0.67			

Habitat specificity was low. From the 57 bird species used in the analyses, open habitats had four (7%) habitat specific species, while the forested habitats had 15 (26.3%), of which only one (1.7%) was specific to botanically near-pristine forest and none were specific to degraded forest. The specificity index was similar across the disturbance categories (χ^2^ = 62, *P =* 0.261) and the majority of bird species (64.4%) occurred across the entire disturbance gradient.

**Fig 3:**
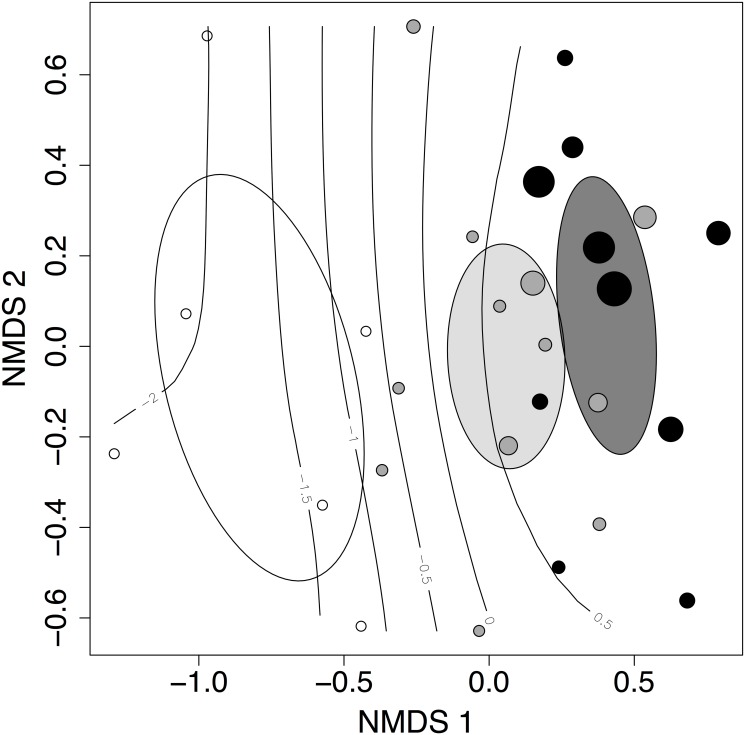
A non-metric multidimensional scaling (NMDS) ordination of the bird assemblages in near pristine forests (black), degraded forests (grey) and open habitats (white). Ellipses are 95% confidence intervals of treatment-level centroids and points are census plots. There was a significant difference between forest bird assemblages and open land assemblages (P = 0.001), but not between near-pristine and degraded forests. The contours indicate the leaf area index and the size of the circle is proportional to the distance to open habitat.

There was no significant difference in community composition between forest habitats (near-pristine and degraded forests; *P* = 0.12). However, community composition varied significantly between open and forested habitats (*P =* 0.001; Fig. 3). We identified distance to open habitat (P = 0.001; Fig. 3) and leaf area index (P = 0.001; Fig. 3) as the best predictors of change in bird composition.

### Extirpated birds

A total of 185 resident bird species was recorded between 1998 and 2012. Of these, 37 (20%) were not observed in 2012. A further 69 species were inferred to have occurred in Mengsong in the past. These species were probably extirpated prior to 1998. Thus, we estimate that a total of 106 species (41.7%; S2 Table) have been extirpated from Mengsong.

**Fig 4:**
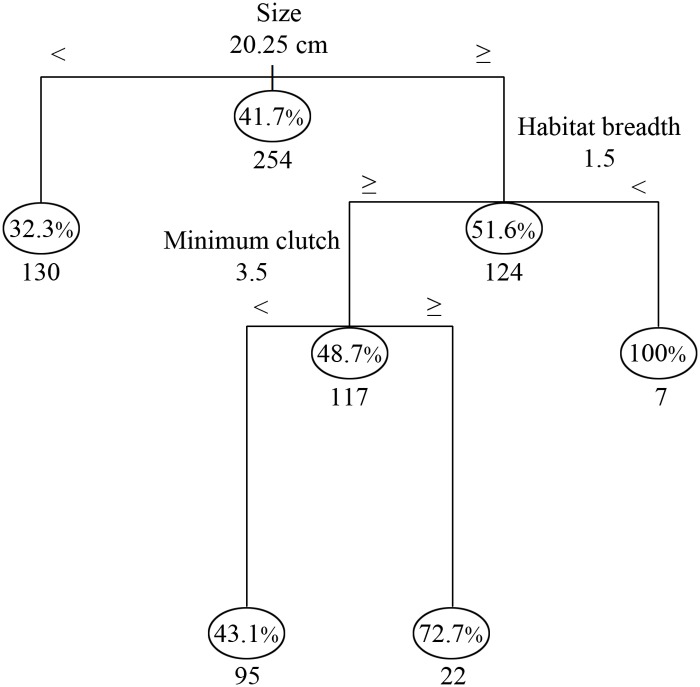
Classification tree showing extinction probabilities of birds in Mengsong based on life history and ecological traits. Body size, habitat breadth and minimum clutch size emerged as the only important factors in our analysis. The percentage in ovals refer to the probability of extinction and numbers below the ovals are the number of species at each node. Species with higher extinction risk are to the right of each branch point.

**Fig 5:**
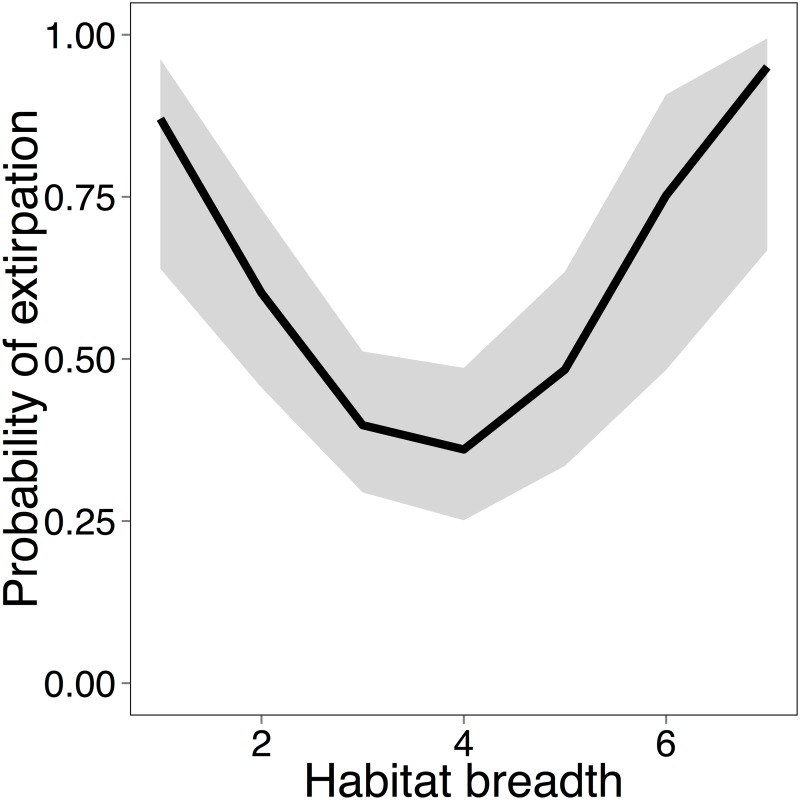
Extirpation probability of large birds (>20.25 cm) as a function of habitat breadth in Mengsong. The line is the prediction of the model fitted to the data with 95% confidence interval.

In the analysis of factors determining extirpation probability (EP), body size, habitat breadth and minimum clutch size were selected in the optimal classification tree, which had four terminal nodes (Fig. 4). Body size was the splitting factor at first node. Over half (51.6%) of the species that were ≥20.25 cm were extirpated, and the EP for birds in Mengsong increased with body size (Dev_(1,252)_ = 11.22; *P* = 0.0008). Further, for large birds (≥20.25 cm), EP had a ‘U’ shaped distribution with increasing habitat breadth (Dev_(1,121)_ = 15.36, *P* < 0.0001; Fig. 5). Thus, large birds with the lowest and highest habitat breadth had high EP. Large birds that had a habitat breadth <1.5 had a 100% EP (Fig. 4). Extirpation probability for large birds with higher habitat breadth (>1.5) increased with minimum clutch size (Dev_(1,109)_ = 7.94, *P* < 0.004) and birds that had a minimum clutch size >3.5 had a 72.7% probability of extirpation (Fig. 4).

### Hunting Pressure

A total of 59 hunters were sighted in 107.5 hours across all the three disturbance categories in Mengsong. The number of hunters sighted on trails did not differ between disturbance categories (χ^2^ = 1.79, *P =* 0.41).

## Discussion

Our study strongly suggests that at least 20% and probably over 40% of the bird species that formerly occurred in the Mengsong landscape have been extirpated, including most of the larger, more charismatic birds and seven out of eight species that are of conservation concern (Table 1) [41].

Extirpation rates can be inflated if recent surveys fail to detect extant species. However, we believe that our bird inventories were most probably complete. First, we spent an extended period in the field (>6 person months over a two year period) and covered the landscape very thoroughly. Second, most extirpated species were highly conspicuous, large birds, such as hornbills (Bucerotidae), parakeets (Psittacidae), woodpeckers (Picidae), bee-eaters (Meropidae) and starlings (Sturnidae) that, if present, are normally detected within a short period in this kind of landscape. Furthermore, no more species have been added to our list in the two years since our survey was conducted, despite considerable interest in the area among Chinese birders, and interviews with knowledgeable local villagers have also failed to add any species [34]. Finally, even if a few rare species were overlooked, the impact on our results would be trivial and would not alter the overall conclusions.

Although a distinct assemblage of open area birds was identified, there was no significant difference in composition among forest habitats and habitat specificity was extremely low. Sixty-four percent of the bird species were observed across the entire disturbance gradient, and among the forest birds, 98% were found in both botanically near-pristine and degraded forests. A probable explanation of this pattern is that a majority of the large, primary forest specialists and birds that prefer open landscapes have been extirpated (Figs. 4 and 5). For example, almost all bird species from families such as Bucerotidae (hornbills), Trogonidae (trogons), Psittacidae (parakeets and hanging-parrots) and Picidae (woodpeckers) have been extirpated. In particular, the family Picidae is highly speciose in the region, but is also known to be sensitive to human disturbance [54,55]. In Mengsong, the White-browed piculet (*Sasia ochracea*; body size 9 cm) is the only extant woodpecker from 12 species that were inferred to occur in the area. Interestingly, large habitat generalists also suffered high extirpation probabilities. Human-modified habitats, such as paddy fields, plantations and reservoirs around villages were almost completely empty of larger birds, and most of the normally common open-land birds belonging to the families Corvidae (crows, jays and magpies), Ardeidae (egrets and herons), Meropidae (bee-eaters) and Sturnidae (mynas and starlings) have been extirpated. Finally, we found that hunting pressure in Mengsong was extremely high, despite the fact that gun ownership has been illegal in China since 1996 [35]. In addition to people with guns, we frequently encountered hunters employing nets and snares. The use of nets, in particular, indicates that hunters are actively harvesting even the smallest birds, which they barbeque on skewers [34]. We suggest that hunting is the most likely explanation of most, if not all, bird extirpations in Mengsong.

In the absence of any evidence of an invasive predator or emergent disease, only area effects and climate change could potentially be suggested as alternative causes of extirpations. However, neither are adequate explanations for the bird extirpations in Mengsong. Our inferred checklist was based on Sino-Russian bird expeditions in the region that were conducted within the last 70 years (1955–70) [45–50], thus bird extirpations in Mengsong were relatively recent. Moreover, as our data show, most bird species in this region have medium to wide distributions and the area is topographically complex. These factors should lessen any impacts of climate change, as species should be pre-adapted to a wide range of environmental conditions and, if necessary, can move up-slope to track climate envelopes [56–59]. As far as our knowledge goes, so far no one has attributed the extirpation of any bird species to climate change, although several authors predict extirpations mostly of high-elevation species [59,60]. Thus, it seems unlikely that climate change could have been an important cause of such widespread extirpation in Mengsong. Landsat images indicate that since 1988 the forest area in Mengsong has remained approximately stable or even slightly increased, so deforestation cannot explain the recorded extirpations. It is possible that earlier deforestation contributed to some of the inferred extirpations. However, the area of forest habitat in Mengsong is ~50,000 ha, which is considerably larger than the home range of any extirpated species (or near-relative) and ought to be large enough to support populations of most species. Moreover, large birds like hornbills and imperial pigeons are known to persist in landscapes with small forest fragments, production forests, and agroforestry systems in the absence of hunting [61–63]. Lastly, 41% of the extirpated species in Mengsong were open-land species. For comparison, in heavily urbanized Singapore, which is also affected by invasive bird species but not modern hunting, only 6% of extirpated species were open-land species [64].

Though hunting is known to be a substantial threat to species persistence, studies focusing on habitat loss and fragmentation often do not distinguish between species lost through area effects and those potentially lost through other causes, including hunting (but see [26,65]). We demonstrated that body size best-predicted bird extirpations in Mengsong. Earlier authors have suggested that large home-range requirements might contribute to the elimination of large birds in fragmented habitats (e.g., [66]). However, large birds are also preferred quarry, and tend to have smaller population sizes and lower reproductive outputs in comparison to smaller birds (i.e. older age to maturity, small clutch size and small number of clutches per year), which make them vulnerable to hunting [67]. It is possible that the role of hunting has not been adequately considered in earlier studies on area effects and fragmentation in the tropics.

Our results indicate that the recently established Bulong Nature Reserve does not support a bird community of any notable value to conservation, despite the fact that it supports a substantial area of natural forest habitat and is located in one of the planet’s most biodiverse regions [68]. We suspect that the mammal fauna is in a similar impoverished state, as mammal sightings were rare and wild meat is often imported from the neighbouring villages in Myanmar [34]. Other vertebrates such as large snakes (e.g. *Naja kaouthia*, *Ptyas korros*), large geckos (e.g. *Gekko gekko*) and many smaller mammals were often observed as an additive in the local liquor, and large frogs (e.g. Dicroglassidae and Megophryidae, which are mostly wild caught) are often observed in local restaurants. It is important to note that Bulong Nature Reserve was only recently designated as a nature reserve and hence the park authorities cannot be held responsible for the current situation. However, unless there is a concerted effort by the authorities to control hunting and restore animal populations, it is clear the reserve will fall well short of its potential to conserve vertebrate biodiversity. Even if hunting is effectively controlled within the reserve, natural recolonisation may be difficult or impossible for many species, because wildlife populations in neighboring areas of Myanmar and Xishuangbanna are probably also heavily depleted [26,69–71]. Making natural recolonisation a realistic possibility will require controlling hunting across the broader landscape.

The local economy in Xishuangbanna is well developed due to the production of cash crops, such as tea at higher elevations and rubber in lower areas. Domestic protein sources are also widely available. However, ethnic groups in the area covet the tradition of hunting and like to eat wild meat when they can get it. As has occurred throughout SE Asia, traditional hunting technologies, such as bows or blowpipes, has been replaced with shotguns and mist-nets that require comparatively little skill to use. Reports from across SE Asia indicate that more and more sites are becoming defaunated. For example, 1) in Vietnam over-hunting has extirpated 13 large vertebrates in the last 40 years [72]. 2) Across a large swath of northern Myanmar hunting has extirpated species and is depleting populations of others [71]. 3) In remote forests of central Borneo hunting has reduced populations of large mammals and hornbills [23]. 4) At Lambir in NW Borneo 20% of mammal species and 50% of bird species over 1 kg have been extirpated by hunting since 1984 [73]. 5) In the Nagaland state of NE India an estimated 120,000 Amur falcons (*Falco amurensis*) were harvested annually until recently [74], and an estimated 16,500 wild birds and mammals were sold annually at a single daily market, resulting in the extirpation of many culturally important species to the Naga [69]. Similar reports from other tropical regions suggest that an ever-increasing number of tropical forests across the globe are empty (e.g., [23,25,26,28,75–78]). It is worth noting that most of this hunting is for bush-meat [24,79]. Hunting to supply the international trade in wildlife and wildlife products may be critically impacting the populations of some species, but far greater numbers of animals are killed for domestic consumption. Across much of the tropical forest biome, hunting may now surpass the importance of habitat loss in determining the persistence of both birds and mammals.

This observation implies that simply extending tropical protected area networks is unlikely to deliver desired conservation outcomes, at least with respect to biodiversity. In China, for example, although the number of nature reserves increased from around 25 to 1750 between 1980 and 2000 [80], bringing 15% of the land mass under protection, and an average of three million hectares has been reforested every year since 2000, hunting threatens to undo any positive gains achieved through increased habitat protection and restoration [79,81]. A third of these reserves have no clear boundaries or management teams and skins of endangered animals, such as snow leopards, are displayed conspicuously in tourist shops around some reserves (see [80]). Although the situation may be changing in some places, hunting is still probably the most serious threat to biodiversity conservation within Chinese protected areas [79], and the situation appears to be similar in many tropical developing nations across the world [5,12].

Previous authors have pointed out that even “paper parks” have conservation value [82] and arguably many defaunated reserves, including the reserve we studied here, are considerably better off than “paper parks”. Such forests may continue to harbor other elements of biodiversity, including plants, arthropods, and meso- and micro-organisms, although the loss or impairment of ecosystem functions performed by extirpated species is likely to lead to a further erosion of biodiversity over time [27,73,83]. They may also support other ecosystem services, such as watershed protection and local climate amelioration. Nonetheless, defaunated reserves clearly fail on the important objective of providing a sanctuary for imperiled wildlife.

With so much of the tropical forest biome already defaunated or in the process of being defaunated, the establishment of new reserves that, more often than not, are ineffective at controlling hunting is a minimal gain for biodiversity conservation. Establishing corridors between reserves that are devoid of the animals that would use them is even less likely to be a benefit to conservation. Importantly, it is clear that many endangered species are capable of persisting in degraded forests and landscapes with fragmented forests, plantations, and agroforestry systems, when they are not exposed to hunting [84,85]. Hence, contrary to the current emphasis on extending the conservation estate, we question whether more might be achieved for tropical conservation if greater investment and effort were channeled into controlling hunting within the current protected area estate and improved wildlife management in the broader landscape.

## Supporting Information

S1 Table Mean ± standard deviation for vegetative and structural habitat characteristics of three habitat types in Bulong Nature Reserve (BNR), China. Paired t-tests were used to compare means. Asterisks indicate the level of significance: *P ≤ 0.05, **P < 0.001, ***P < 0.0001 and NS is not significant (P > 0.05). Significance levels were adjusted using Bonferroni correction for multiple comparisons. Aspect was cosine transformed and basal area was log-transformed before analysis. Near-pristine were botanically pristine forests, degraded were secondary re-growth forests, and open were tea plantations or degraded grasslands.
(DOCX)

S2 Table Summary information of species recorded and inferred to occur in Bulong Nature reserve (BNR), including their taxonomic information, extirpation status, and life-history and ecological traits. Body size (centimetres); Forest habitat (P = prefer forest habitat, NP = no preference); Disturbed habitat use (Y = yes, N = no); Primary diet type (inver = invertebrates, VertFish = vertebrates, frug = fruit; nec = nectar, gran = seed); Nest substrate/level (G = ground, NG = not ground); Range (R = restricted distribution, M = medium distribution, W = wide distribution); NA means no data.
(DOCX)

S3 Table Species detected at each plot. (DOCX)
