# Supplementary material for: Yet Another Empty Forest: Considering the Conservation Value of a Recently Established Tropical Nature Reserve
Source: PLoS One. 2015 Feb 10;10(2):e0117920. doi: 10.1371/journal.pone.0117920 (PMC4323245; doi:10.1371/journal.pone.0117920)
Supplement: S1 Table — Paired t-tests were used to compare means. Asterisks indicate the level of significance: *P ≤ 0.05, **P < 0.001, ***P < 0.0001 and NS is not significant (P > 0.05). Significance levels were adjusted using Bonferroni correction for multiple comparisons. Aspect was cosine transformed and basal area was log-transformed before analysis. Near-pristine were botanically pristine forests, degraded were secondary re-growth forests, and open were tea plantations or degraded grasslands. (DOCX) [file pone.0117920.s001.docx]

**Table S1.** **Mean ± standard deviation for vegetative and structural habitat characteristics of three habitat types in Bulong Nature Reserve (BNR), China.** Paired t-tests were used to compare means. Asterisks indicate the level of significance: **P* ≤ 0.05, ***P <* 0.001, ****P <* 0.0001 and NS is not significant (P > 0.05). Significance levels were adjusted using Bonferroni correction for multiple comparisons. Aspect was cosine transformed and basal area was log-transformed before analysis. Near-pristine were botanically pristine forests, degraded were secondary re-growth forests, and open were tea plantations or degraded grasslands.

| Habitat characteristic | Near-pristine | Degraded | Open |  | t-value |  |
| --- | --- | --- | --- | --- | --- | --- |
|  |  |  |  | Near-pristine versus degraded | Near-pristine versus open | Degraded versus open |
| Slope angle | 29.7±5.1 | 26.9±3.5 | 26.8±7.8 | 1.49^NS^ | 0.81^NS^ | 0.01^NS^ |
| Aspect | -0.28±0.7 | 0.05±0.6 | -0.47±0.5 | -1.27^NS^ | 0.61^NS^ | 1.89^NS^ |
| Elevation | 1618±222 | 1606±217 | 1651±164 | 0.12^NS^ | -0.35^NS^ | -0.49^NS^ |
| Leaf area index(LAI) | 3.62±0.2 | 3.13±0.5 | 0.52±0.5 | 3.15* | 15.03*** | 11.22*** |
| Basal area | 8.63±2.14 | 5.61±1.70 | 0.53±0.55 | 3.64* | 12.54*** | 9.13*** |
| Distance to open land | 617±389 | 252±232 | 0±0 | 2.96* | 25.95*** | 20.02*** |
