# Supplementary material for: Yet Another Empty Forest: Considering the Conservation Value of a Recently Established Tropical Nature Reserve
Source: PLoS One. 2015 Feb 10;10(2):e0117920. doi: 10.1371/journal.pone.0117920 (PMC4323245; doi:10.1371/journal.pone.0117920)
Supplement: S2 Table — Body size (centimetres); Forest habitat (P = prefer forest habitat, NP = no preference); Disturbed habitat use (Y = yes, N = no); Primary diet type (inver = invertebrates, VertFish = vertebrates, frug = fruit; nec = nectar, gran = seed); Nest substrate/level (G = ground, NG = not ground); Range (R = restricted distribution, M = medium distribution, W = wide distribution); NA means no data. (DOCX) [file pone.0117920.s002.docx]

**Table S2. Summary information of species recorded and inferred to occur in Bulong Nature reserve (BNR), including their taxonomic information, extirpation status, and life-history and ecological traits.** Body size (centimetres); Forest habitat (P=prefer forest habitat, NP=no preference); Disturbed habitat use (Y=yes, N=no); Primary diet type (inver = invertebrates, VertFish = vertebrates, frug = fruit; nec = nectar, gran = seed); Nest substrate/level (G=ground, NG=not ground); Range (R = restricted distribution, M = medium distribution, W = wide distribution); NA means no data.

| Scientific Name | Extirpation status | Forest habitat | Disturbed habitat use | Habitat breadth | Diet breadth | Primary diet type | Body size | Minimum clutch | Nest substrate | Range |
| --- | --- | --- | --- | --- | --- | --- | --- | --- | --- | --- |
| *Abroscopus albogularis* | Extant | P | Y | 2 | 1 | inver | 9 | 3 | NG | M |
| *Abroscopus superciliaris* | Extant | P | Y | 4 | 1 | inver | 10.5 | 3 | NG | W |
| *Accipiter trivirgatus* | Extant | P | N | 3 | 1 | VertFish | 43 | 2 | NG | W |
| *Accipiter virgatus* | Extant | P | N | 3 | 2 | VertFish | 29.5 | 2 | NG | W |
| *Aceros nipalensis* | Extinct | P | N | 1 | 3 | frug | 117 | 2 | NG | R |
| *Acridotheres cristatellus* | Extinct | NP | Y | 6 | 3 | inver | 26.5 | 4 | NG | R |
| *Acridotheres grandis* | Extant | NP | Y | 7 | 3 | frug | 26 | 4 | NG | M |
| *Acridotheres tristis* | Extinct | NP | Y | 7 | 4 | inver | 26 | 4 | NG | W |
| *Aegithalos concinnus* | Extant | P | Y | 4 | 3 | inver | 11 | 4 | NG | W |
| *Aegithina lafresnayei* | Extant | P | Y | 2 | NA | inver | 16 | 3 | NG | M |
| *Aegithina tiphia* | Extant | NP | Y | 6 | 4 | inver | 13 | 2 | NG | W |
| *Aethopyga gouldiae* | Extant | P | Y | 2 | 2 | nec | 14 | 2 | NG | M |
| *Aethopyga nipalensis* | Extant | P | Y | 2 | 2 | nec | 12 | 2 | NG | M |
| *Aethopyga saturata* | Extant | P | Y | 2 | 4 | nec | 13 | 1 | NG | M |
| *Alauda gulgula* | Extant | NP | Y | 3 | 3 | gran | 17 | 3 | G | W |
| *Alcedo atthis* | Extant | NP | Y | 5 | 2 | VertFish | 17 | 2 | G | W |
| *Alcedo hercules* | Extant | P | Y | 2 | 2 | VertFish | 22.5 | 4 | G | R |
| *Alcedo meninting* | Extant | P | Y | 3 | 2 | VertFish | 16 | 3 | G | W |
| *Alcippe fratercula* | Extant | P | Y | 3 | 4 | frug | 14 | 2 | NG | M |
| *Alcippe poioicephala* | Extant | P | Y | 3 | 3 | inver | 16 | 2 | NG | W |
| *Alophoixus flaveolus* | Extinct | P | N | 1 | 2 | frug | 22 | 2 | NG | R |
| *Alophoixus pallidus* | Extinct | P | N | 2 | 3 | frug | 22 | NA | NG | R |
| *Amandava amandava* | Extant | NP | Y | 4 | 2 | frug | 10 | 5 | NG | W |
| *Amaurornis phoenicurus* | Extant | NP | Y | 6 | 3 | inver | 33 | 3 | G | W |
| *Anorrhinus austeni* | Extinct | P | N | 1 | 3 | frug | 63 | 2 | NG | R |
| *Anthus roseatus* | Extinct | NP | Y | 3 | 2 | inver | 16.5 | 3 | G | W |
| *Anthus rufulus* | Extant | NP | Y | 5 | 1 | inver | 15.5 | 2 | G | W |
| *Apus nipalensis* | Extant | NP | Y | 2 | 1 | inver | 14.5 | 2 | NG | W |
| *Arachnothera longirostra* | Extinct | P | Y | 4 | 3 | nec | 16 | 2 | NG | W |
| *Arachnothera magna* | Extant | P | Y | 3 | 3 | nec | 19 | 2 | NG | M |
| *Arborophila brunneopectus* | Extinct | P | Y | 2 | 4 | NA | 28 | 4 | G | R |
| *Arborophila chloropus* | Extant | P | Y | 3 | 5 | NA | 29 | 3 | G | R |
| *Arborophila rufogularis* | Extinct | P | Y | 2 | 4 | frug | 27 | 3 | G | M |
| *Ardeola bacchus* | Extant | NP | Y | 3 | 3 | VertFish | 48 | 3 | NG | W |
| *Artamus fuscus* | Extinct | NP | Y | 6 | 1 | inver | 17 | 2 | NG | W |
| *Aviceda leuphotes* | Extinct | P | N | 3 | 2 | inver | 32.5 | 2 | NG | W |
| *Bambusicola fytchii* | Extant | NP | Y | 5 | 5 | inver | 34.5 | 3 | G | R |
| *Blythipicus pyrrhotis* | Extinct | P | Y | 4 | 1 | inver | 27.5 | 2 | NG | M |
| *Brachypteryx montana* | Extant | P | N | 2 | 1 | inver | 13 | 3 | NG | W |
| *Bradypterus luteoventris* | Extinct | NP | Y | 4 | 1 | inver | 14 | 3 | NG | M |
| *Bubulcus coromandus* | Extant | NP | Y | 2 | 1 | inver | 50 | 3 | NG | W |
| *Buceros bicornis* | Extinct | P | N | 1 | 3 | frug | 122 | 2 | NG | W |
| *Cacomantis sonneratii* | Extinct | P | Y | 4 | 1 | inver | 23.5 | NA | NG | W |
| *Carduelis ambigua* | Extinct | NP | Y | 4 | NA | gran | 13 | 3 | NG | R |
| *Cecropis striolata* | Extant | NP | Y | 6 | 1 | inver | 18.5 | 2 | NG | W |
| *Celeus brachyurus* | Extinct | NP | Y | 5 | 2 | inver | 25 | 2 | NG | W |
| *Centropus bengalensis* | Extant | NP | Y | 5 | 2 | inver | 38 | 2 | NG | W |
| *Centropus sinensis* | Extant | NP | Y | 5 | 2 | inver | 50 | 2 | NG | W |
| *Ceryle rudis* | Extinct | NP | Y | 6 | 1 | VertFish | 29 | 4 | G | W |
| *Cettia fortipes* | Extant | NP | Y | 2 | 2 | inver | 12 | 3 | NG | M |
| *Chalcoparia singalensis* | Extinct | P | Y | 6 | 2 | nec | 10.5 | 2 | NG | W |
| *Chalcophaps indica* | Extant | P | Y | 3 | 2 | frug | 25 | 2 | NG | W |
| *Chelidorhynx hypoxantha* | Extant | NP | Y | 2 | 1 | inver | 12 | 3 | NG | M |
| *Chloropsis cochinchinensis* | Extant | P | Y | 4 | 4 | inver | 17.5 | 2 | NG | W |
| *Chloropsis hardwickii* | Extant | P | N | 3 | 4 | inver | 19.5 | 2 | NG | M |
| *Chrysococcyx maculatus* | Extinct | P | Y | 5 | 1 | inver | 17 | NA | NG | M |
| *Chrysococcyx xanthorhynchus* | Extinct | P | Y | 5 | 1 | inver | 17 | NA | NG | W |
| *Chrysocolaptes lucidus* | Extinct | NP | Y | 5 | 2 | inver | 30.5 | 4 | NG | W |
| *Chrysolophus amherstiae* | Extinct | NP | Y | 4 | 4 | gran | 150 | 6 | G | R |
| *Chrysomma sinense* | Extinct | NP | Y | 3 | 1 | inver | 18 | 3 | NG | W |
| *Cinnyris jugularis* | Extinct | P | Y | 7 | 2 | nec | 11.5 | 2 | NG | W |
| *Cissa chinensis* | Extant | P | N | 3 | 4 | inver | 39 | 3 | NG | W |
| *Cisticola juncidis* | Extinct | NP | Y | 3 | 2 | inver | 11 | 2 | NG | W |
| *Cochoa viridis* | Extant | P | N | 2 | 2 | inver | 27 | 2 | NG | M |
| *Columba hodgsonii* | Extant | P | N | 2 | 2 | frug | 38 | 3 | NG | M |
| *Copsychus malabaricus* | Extinct | NP | Y | 4 | 5 | inver | 25 | 4 | G | W |
| *Copsychus saularis* | Extant | NP | Y | 7 | 5 | inver | 20 | 3 | G | W |
| *Coracias benghalensis* | Extinct | NP | Y | 5 | 4 | inver | 32.5 | 3 | NG | W |
| *Coracina macei* | Extinct | P | Y | 4 | 3 | inver | 29 | 2 | NG | W |
| *Lalage melaschistos* | Extinct | P | Y | 4 | 3 | inver | 23.5 | 2 | NG | W |
| *Corvus macrorhynchos* | Extinct | NP | Y | 5 | 5 | frug | 53 | 3 | NG | W |
| *Corvus splendens* | Extant | NP | Y | 4 | 5 | frug | 42 | 3 | NG | W |
| *Culicicapa ceylonensis* | Extant | P | Y | 5 | 1 | inver | 12.5 | 2 | NG | W |
| *Cutia nipalensis* | Extinct | P | N | 3 | 1 | inver | 18 | NA | NG | M |
| *Cyornis glaucicomans* | Extinct | P | Y | 5 | 1 | inver | 14.5 | 3 | NG | W |
| *Delichon nipalense* | Extant | P | N | 5 | 1 | inver | 12 | 3 | NG | R |
| *Dendrocitta formosae* | Extant | P | N | 5 | 5 | inver | 38 | 3 | NG | W |
| *Dendrocopos atratus* | Extinct | P | Y | 2 | 1 | inver | 21 | 4 | NG | R |
| *Dendrocopos canicapillus* | Extinct | NP | Y | 5 | 1 | inver | 14 | 3 | NG | W |
| *Dendrocopos hyperythrus* | Extinct | P | Y | 2 | 1 | inver | 22 | 4 | NG | M |
| *Dicaeum chrysorrheum* | Extant | P | Y | 5 | 2 | inver | 10 | 2 | NG | W |
| *Dicaeum cruentatum* | Extinct | P | Y | 5 | 3 | frug | 8.5 | 2 | NG | W |
| *Dicaeum ignipectus* | Extant | P | Y | 2 | 2 | frug | 8.5 | 2 | NG | W |
| *Dicaeum melanoxanthum* | Extinct | P | N | 2 | NA | inver | 13 | NA | NA | M |
| *Dicaeum minullum* | Extant | P | Y | 4 | 3 | nec | 8 | 2 | NG | W |
| *Dicrurus aeneus^a^* | Extinct | P | Y | 4 | 1 | inver | 22.5 | 2 | NG | W |
| *Dryonastes chinensis* | Extant | P | Y | 4 | 3 | frug | 28 | 3 | NG | R |
| *Ducula aenea* | Extant | P | Y | 3 | 1 | frug | 45 | 1 | NG | W |
| *Ducula badia* | Extant | P | Y | 2 | 1 | frug | 48 | 1 | NG | W |
| *Egretta garzetta* | Extinct | NP | Y | 3 | 3 | VertFish | 60 | 3 | NG | W |
| *Egretta intermedia* | Extinct | NP | Y | 3 | 2 | VertFish | 68 | 3 | NG | W |
| *Elanus caeruleus* | Extant | NP | Y | 4 | 2 | VertFish | 33.5 | 3 | NG | W |
| *Emberiza fucata* | Extant | NP | Y | 5 | 2 | inver | 15.5 | 3 | G | W |
| *Enicurus leschenaulti* | Extant | P | N | 3 | 1 | inver | 28 | 2 | G | W |
| *Enicurus schistaceus* | Extant | P | N | 3 | 1 | inver | 23.5 | 2 | G | W |
| *Enicurus scouleri* | Extant | P | N | 3 | 1 | inver | 13.5 | 2 | G | W |
| *Erpornis zantholeuca* | Extant | P | N | 2 | 2 | inver | 13 | 2 | NG | W |
| *Falco peregrinus* | Extinct | NP | Y | 7 | 1 | VertFish | 44 | 3 | NG | W |
| *Falco severus* | Extinct | NP | Y | 7 | 2 | inver | 28.5 | 2 | NG | W |
| *Ficedula hyperythra* | Extinct | P | N | 2 | 1 | inver | 12 | 4 | G | W |
| *Ficedula sapphira* | Extant | P | N | 2 | 1 | inver | 11 | 4 | G | M |
| *Ficedula westermanni* | Extinct | P | N | 3 | 1 | inver | 12 | 3 | G | W |
| *Fulvetta manipurensis* | Extant | P | Y | 2 | 3 | inver | 12 | NA | NA | R |
| *Gallus gallus* | Extant | P | Y | 3 | 5 | gran | 71 | 4 | G | W |
| *Gampsorhynchus torquatus* | Extinct | P | Y | 1 | 1 | inver | 24 | 3 | NG | R |
| *Garrulax leucolophus* | Extinct | P | Y | 3 | 4 | frug | 29 | 2 | NG | M |
| *Garrulax monileger* | Extinct | P | N | 2 | 2 | inver | 29 | 3 | NG | M |
| *Ianthocincla pectoralis* | Extinct | P | N | 2 | 2 | inver | 31 | 3 | NG | W |
| *Garrulus glandarius* | Extinct | P | Y | 2 | 4 | inver | 32.5 | 3 | NG | W |
| *Gracupica nigricollis* | Extinct | NP | Y | 6 | 3 | inver | 29 | 3 | NG | M |
| *Harpactes erythrocephalus* | Extinct | P | Y | 2 | 2 | inver | 33 | 2 | NG | W |
| *Harpactes oreskios* | Extinct | P | Y | 2 | 1 | inver | 29 | 2 | NG | W |
| *Hemipus picatus* | Extant | P | Y | 3 | 1 | inver | 13.5 | 2 | NG | W |
| *Hemixos flavala* | Extant | P | Y | 3 | 4 | frug | 20.5 | 2 | NG | W |
| *Hierococcyx sparverioides* | Extinct | P | Y | 3 | 1 | inver | 40 | NA | NG | W |
| *Hirundo rustica* | Extant | NP | Y | 9 | 2 | inver | 15 | 4 | NG | W |
| *Hodgsonius phaenicuroides* | Extant | NP | Y | 6 | 2 | inver | 18 | 2 | NG | W |
| *Hypogramma hypogrammicum* | Extinct | P | N | 2 | 2 | nec | 14.5 | 2 | NG | W |
| *Hypothymis azurea* | Extinct | P | Y | 6 | 1 | inver | 17 | 2 | NG | W |
| *Hypsipetes leucocephalus* | Extant | P | Y | 3 | 4 | frug | 25 | 2 | NG | W |
| *Ictinaetus malayensis* | Extant | P | Y | 3 | 1 | VertFish | 76 | 1 | NG | W |
| *Irena puella* | Extinct | P | N | 2 | 2 | frug | 25.5 | 2 | NG | W |
| *Ixos mcclellandii* | Extant | P | Y | 2 | 4 | frug | 22.5 | 2 | NG | M |
| *Lanius collurioides* | Extinct | NP | Y | 3 | 2 | inver | 20 | 3 | NG | W |
| *Lanius schach* | Extant | NP | Y | 3 | 3 | inver | 26 | 3 | NG | W |
| *Lanius tephronotus* | Extant | NP | Y | 4 | 4 | inver | 24 | NA | NA | W |
| *Leioptila desgodinsi* | Extant | P | Y | 2 | 4 | inver | 22.5 | NA | NA | M |
| *Leucodioptron canorum* | Extant | NP | Y | 5 | 1 | inver | 22.5 | 2 | NG | W |
| *Liocichla ripponi* | Extant | P | Y | 3 | 3 | frug | 22 | 2 | NG | R |
| *Lonchura atricapilla* | Extinct | NP | Y | 4 | 2 | gran | 11 | 4 | NG | W |
| *Lonchura punctulata* | Extant | NP | Y | 4 | 2 | gran | 12 | 4 | NG | W |
| *Lonchura striata* | Extant | NP | Y | 4 | 2 | gran | 11 | 3 | NG | W |
| *Lophura nycthemera* | Extant | P | Y | 2 | 4 | gran | 104 | 4 | G | R |
| *Mixornis gularis* | Extant | P | Y | 4 | 1 | inver | 13 | 2 | NG | W |
| *Macropygia unchall* | Extant | P | Y | 4 | 2 | gran | 39.5 | 1 | NG | W |
| *Megaceryle lugubris* | Extinct | NP | N | 4 | 1 | VertFish | 40 | 4 | G | W |
| *Psilopogon asiatica* | Extant | P | Y | 4 | 3 | frug | 23 | 3 | NG | W |
| *Psilopogon australis* | Extinct | P | Y | 3 | 3 | frug | 17.5 | 3 | NG | W |
| *Psilopogon haemacephala* | Extinct | P | Y | 5 | 3 | frug | 17 | 2 | NG | W |
| *Psilopogon virens* | Extant | P | Y | 3 | 3 | frug | 32.5 | 2 | NG | M |
| *Megalurus palustris* | Extant | NP | Y | 4 | 1 | inver | 26.5 | 3 | NG | W |
| *Melanochlora sultanea^a^* | Extinct | P | N | 3 | 1 | inver | 20.5 | 5 | NG | M |
| *Melophus lathami* | Extant | NP | Y | 4 | 2 | inver | 16.5 | 3 | G | W |
| *Merops orientalis* | Extinct | NP | Y | 7 | 1 | inver | 19.5 | 4 | G | W |
| *Mesia argentauris* | Extant | P | Y | 4 | 3 | frug | 17 | 2 | NG | W |
| *Microhierax melanoleucos* | Extinct | P | Y | 3 | 2 | inver | 19.5 | 3 | NG | W |
| *Milvus migrans* | Extinct | NP | Y | 7 | 2 | VertFish | 58 | 2 | NG | W |
| *Actinodura cyanouroptera* | Extant | P | Y | 2 | 3 | inver | 15 | 2 | G | W |
| *Monticola rufiventris* | Extant | P | N | 3 | NA | inver | 23 | 3 | G | W |
| *Monticola solitarius* | Extant | NP | Y | 4 | 4 | inver | 22 | 3 | NG | W |
| *Muscicapa muttui* | Extinct | P | N | 2 | 1 | inver | 14.5 | 4 | NG | W |
| *Myiomela leucura* | Extant | P | N | 2 | 2 | inver | 18.5 | 2 | G | W |
| *Myophonus caeruleus* | Extant | P | N | 4 | 3 | inver | 33 | 2 | G | W |
| *Napothera epilepidota* | Extant | P | N | 2 | 1 | inver | 11 | 2 | G | W |
| *Niltava macgrigoriae* | Extinct | P | N | 2 | 1 | inver | 13.5 | 3 | G | M |
| *Niltava vivida* | Extant | P | N | 1 | 1 | inver | 18.5 | NA | NA | R |
| *Nyctyornis athertoni* | Extant | P | N | 4 | 1 | inver | 35 | 4 | G | W |
| *Oriolus chinensis* | Extinct | P | Y | 5 | 2 | inver | 25.5 | 2 | NG | W |
| *Oriolus tenuirostris* | Extant | P | Y | 3 | 2 | inver | 24.5 | 2 | NG | M |
| *Orthotomus sutorius* | Extant | NP | Y | 5 | 1 | inver | 12 | 2 | NG | W |
| *Parus minor* | Extant | NP | Y | 7 | 4 | inver | 14 | 3 | G | W |
| *Parus spilonotus* | Extant | P | N | 2 | 2 | inver | 14.5 | 4 | G | M |
| *Passer montanus* | Extant | NP | Y | 4 | 3 | gran | 14 | 3 | NG | W |
| *Passer rutilans* | Extinct | NP | Y | 2 | 2 | gran | 13.5 | 4 | NG | W |
| *Pavo muticus* | Extinct | P | Y | 2 | 5 | frug | 220 | 3 | G | M |
| *Pellorneum ruficeps* | Extant | P | Y | 4 | 2 | inver | 17 | 2 | G | W |
| *Pericrocotus flammeus* | Extant | P | Y | 4 | 1 | inver | 20 | 2 | NG | W |
| *Pericrocotus roseus* | Extinct | P | Y | 3 | 1 | inver | 18.75 | 2 | NG | W |
| *Pericrocotus solaris* | Extant | P | Y | 4 | 1 | inver | 18 | 3 | NG | W |
| *Phaenicophaeus tristis* | Extant | P | Y | 4 | 2 | inver | 56 | 2 | NG | W |
| *Phyllergates cucullatus* | Extant | P | Y | 2 | 1 | inver | 11.5 | 3 | NG | W |
| *Pica pica* | Extinct | NP | Y | 2 | 3 | inver | 45 | 5 | NG | W |
| *Picumnus innominatus* | Extinct | P | Y | 4 | 1 | inver | 10 | 2 | NG | W |
| *Picus canus* | Extinct | NP | Y | 5 | 2 | inver | 32 | 4 | NG | W |
| *Picus chlorolophus* | Extinct | P | Y | 3 | 2 | inver | 26.5 | 2 | NG | W |
| *Picus flavinucha* | Extinct | P | Y | 3 | 1 | inver | 33 | 2 | NG | W |
| *Picus vittatus* | Extinct | NP | Y | 6 | 2 | inver | 30 | 3 | NG | W |
| *Pitta cyanea* | Extinct | P | N | 2 | 1 | inver | 22 | 2 | G | M |
| *Pitta nipalensis* | Extinct | P | N | 3 | 1 | inver | 24 | 3 | G | R |
| *Pitta oatesi* | Extant | P | N | 2 | 1 | inver | 23 | 2 | NG | M |
| *Pitta phayrei* | Extinct | P | N | 1 | 1 | inver | 22 | 4 | G | R |
| *Pnoepyga pusilla* | Extant | P | Y | 2 | 2 | inver | 8.5 | 2 | G | W |
| *Polyplectron bicalcaratum* | Extinct | P | Y | 2 | 4 | gran | 66 | 2 | G | R |
| *Pomatorhinus gravivox* | Extinct | NP | Y | 3 | 4 | inver | 23.5 | 3 | G | M |
| *Pomatorhinus hypoleucos* | Extant | P | N | 2 | 3 | inver | 26.5 | 2 | G | M |
| *Pomatorhinus ochraceiceps* | Extinct | P | N | 1 | 3 | inver | 23 | 3 | G | R |
| *Pomatorhinus ruficollis* | Extant | P | Y | 3 | 3 | inver | 18 | 2 | G | W |
| *Porzana fusca* | Extant | NP | Y | 5 | 3 | inver | 24 | 3 | G | W |
| *Prinia flaviventris* | Extant | NP | N | 4 | 1 | inver | 13.5 | 3 | NG | W |
| *Prinia hodgsonii* | Extant | NP | Y | 5 | 1 | inver | 11 | 3 | NG | W |
| *Prinia inornata* | Extant | NP | Y | 6 | 1 | inver | 14.5 | 3 | NG | W |
| *Prinia rufescens* | Extinct | P | Y | 4 | 1 | inver | 11.5 | 3 | NG | W |
| *Prinia superciliaris* | Extant | NP | Y | 3 | 1 | inver | 18 | 3 | NG | M |
| *Psarisomus dalhousiae* | Extant | P | N | 2 | 5 | inver | 25.5 | 3 | NG | W |
| *Pseudominla castaneceps* | Extant | P | Y | 2 | 4 | inver | 11 | 3 | G | M |
| *Psittacula alexandri* | Extinct | NP | Y | 5 | 3 | frug | 35 | 3 | NG | W |
| *Psittacula derbiana* | Extinct | P | N | 3 | 2 | frug | 43 | NA | NA | R |
| *Psittacula finschii* | Extinct | NP | Y | 5 | 2 | frug | 38 | 3 | NG | R |
| *Psittiparus gularis* | Extant | P | Y | 2 | 3 | inver | 17 | 2 | NG | W |
| *Pterorhinus sannio* | Extant | NP | Y | 5 | 3 | frug | 23 | 3 | NG | M |
| *Pteruthius aeralatus* | Extant | P | N | 3 | 1 | inver | 17 | 2 | NG | W |
| *Pteruthius intermedius* | Extant | P | N | 2 | 1 | inver | 11.5 | 2 | NG | M |
| *Pteruthius melanotis* | Extant | P | N | 2 | 1 | inver | 11.5 | 2 | NG | M |
| *Pycnonotus atriceps* | Extant | P | N | 2 | 1 | frug | 18 | 2 | NG | W |
| *Pycnonotus aurigaster* | Extant | NP | Y | 5 | 5 | frug | 20 | 2 | NG | W |
| *Pycnonotus flavescens* | Extant | NP | Y | 4 | 3 | frug | 21.5 | 2 | NG | M |
| *Pycnonotus flaviventris* | Extant | P | Y | 3 | 1 | frug | 19 | 2 | NG | W |
| *Pycnonotus jocosus* | Extant | NP | Y | 5 | 4 | frug | 19.5 | 2 | NG | W |
| *Pycnonotus xanthorrhous* | Extant | NP | Y | 5 | 5 | frug | 20 | 2 | NG | M |
| *Rhipidura albicollis* | Extant | P | Y | 3 | 1 | inver | 19 | 2 | NG | W |
| *Phoenicurus fuliginosus* | Extinct | NP | Y | 5 | 4 | inver | 15 | 3 | G | W |
| *Sarcogyps calvus* | Extinct | NP | Y | 5 | 1 | VertFish | 83 | 1 | NG | W |
| *Sasia ochracea* | Extant | P | Y | 5 | 1 | inver | 9 | 2 | NG | M |
| *Saxicola caprata* | Extinct | NP | Y | 5 | 1 | inver | 14 | 3 | NG | W |
| *Saxicola ferreus* | Extant | NP | Y | 8 | 2 | inver | 14 | 4 | G | W |
| *Schoeniparus dubius* | Extant | P | Y | 4 | 4 | inver | 14.5 | 2 | G | R |
| *Seicercus castaniceps* | Extant | P | Y | 2 | 1 | inver | 10 | 3 | G | W |
| *Serilophus lunatus* | Extant | P | N | 2 | 3 | inver | 16.5 | 4 | NG | W |
| *Sitta cinnamoventris* | Extant | P | N | 3 | 2 | inver | 13 | 2 | NG | W |
| *Sitta frontalis* | Extant | P | Y | 4 | 2 | inver | 13 | 3 | NG | W |
| *Sitta magna^a^* | ? | P | N | 2 | 2 | inver | 19.5 | 3 | NG | M |
| *Sitta nagaensis* | Extant | P | N | 3 | 2 | inver | 13 | 2 | NG | R |
| *Spilornis cheela* | Extant | P | N | 3 | 1 | VertFish | 65 | 1 | NG | W |
| *Spizixos canifrons* | Extant | NP | Y | 3 | 3 | gran | 21.5 | 2 | NG | R |
| *Stachyridopsis ambigua* | Extinct | NP | Y | 4 | 3 | inver | 12 | 3 | NG | M |
| *Stachyridopsis chrysaea* | Extant | P | N | 2 | 1 | inver | 11 | 3 | G | W |
| *Stachyridopsis ruficeps* | Extinct | P | Y | 2 | 3 | inver | 12.5 | 3 | NG | W |
| *Stachyris nigriceps* | Extant | P | Y | 2 | 1 | inver | 13 | 2 | G | W |
| *Staphida castaniceps* | Extant | P | Y | 3 | 3 | frug | 12.5 | 2 | G | M |
| *Streptopelia orientalis* | Extant | NP | Y | 3 | 1 | gran | 32 | 2 | NG | W |
| *Streptopelia tranquebarica* | Extant | NP | Y | 4 | 2 | gran | 24 | 3 | NG | W |
| *Sturnia malabarica* | Extinct | NP | N | 3 | 3 | frug | 19.5 | 3 | NG | W |
| *Sturnia pagodarum* | Extant | NP | Y | 4 | 3 | frug | 20 | NA | NA | W |
| *Syrmaticus humiae* | Extinct | NP | Y | 4 | 4 | NA | 91 | 6 | G | R |
| *Tephrodornis virgatus* | Extinct | P | Y | 5 | 1 | inver | 18.5 | 2 | NG | W |
| *Terpsiphone paradisi* | Extinct | NP | Y | 6 | 2 | inver | 21.5 | 2 | NG | W |
| *Tesia castaneocoronata* | Extant | P | Y | 2 | 1 | inver | 9 | 2 | NG | M |
| *Tesia olivea* | Extant | P | N | 2 | 1 | inver | 9 | NA | NA | R |
| *Timalia pileata* | Extinct | NP | Y | 4 | 2 | inver | 16 | 2 | NG | M |
| *Treron apicauda* | Extant | P | N | 2 | 2 | frug | 30.5 | 2 | NG | M |
| *Treron curvirostra* | Extant | P | Y | 4 | 2 | frug | 26 | 2 | NG | W |
| *Treron sphenurus* | Extant | P | Y | 3 | 2 | frug | 26 | 2 | NG | W |
| *Turdus dissimilis* | Extant | P | Y | 3 | NA | inver | 23 | 3 | G | R |
| *Turnix suscitator* | Extant | NP | Y | 5 | 4 | inver | 16 | 3 | G | W |
| *Turnix sylvaticus* | Extant | NP | Y | 5 | NA | NA | 13.5 | 4 | G | W |
| *Turnix tanki* | Extinct | NP | Y | 5 | 4 | gran | 17 | 4 | G | W |
| *Upupa epops* | Extant | NP | Y | 3 | 1 | inver | 30 | 5 | NG | W |
| *Urocissa erythrorhyncha* | Extant | P | Y | 5 | 5 | inver | 66.5 | 3 | NG | W |
| *Urocissa whiteheadi* | Extinct | P | Y | 3 | 3 | inver | 39 | 6 | NG | R |
| *Vanellus indicus* | Extinct | NP | Y | 2 | 2 | inver | 33 | 4 | G | W |
| *Yuhina flavicollis* | Extant | P | Y | 2 | 4 | frug | 13 | 2 | G | M |
| *Zoothera citrina* | Extinct | P | N | 4 | 2 | inver | 22 | 2 | NG | W |
| *Zoothera marginata* | Extinct | P | N | 2 | NA | inver | 24 | 3 | NG | M |
| *Zosterops japonicus* | Extant | P | Y | 4 | 3 | inver | 11 | 2 | NG | W |
| *Zosterops palpebrosus* | Extant | P | Y | 5 | 3 | inver | 10.5 | 2 | NG | W |

^a^Unconfirmed sightings
