# Supplementary material for: Yet Another Empty Forest: Considering the Conservation Value of a Recently Established Tropical Nature Reserve
Source: PLoS One. 2015 Feb 10;10(2):e0117920. doi: 10.1371/journal.pone.0117920 (PMC4323245; doi:10.1371/journal.pone.0117920)
Supplement: S3 Table — (DOCX) [file pone.0117920.s003.docx]

**Table S3.** **Species detected at each plot.**

|  | **Near-pristine forest plots** | | | | | | | | | |  | **Degraded forest plots** | | | | | | | | | | |  | **Open habitat plots** | | | | |
| --- | --- | --- | --- | --- | --- | --- | --- | --- | --- | --- | --- | --- | --- | --- | --- | --- | --- | --- | --- | --- | --- | --- | --- | --- | --- | --- | --- | --- |
| Scientific name | 75 | 107 | 189 | 192 | 214 | 217 | 240 | 365 | 398 | 404 | 7 | 49 | 58 | 83 | 90 | 145 | 234 | 259 | 271 | 372 | 381 | 405 | 62 | 119 | 126 | 207 | 357 | 382 |
| *Aegithina lafresnayei* | 0 | 0 | 0 | 0 | 0 | 0 | 0 | 0 | 0 | 0 | 0 | 0 | 0 | 0 | 1 | 0 | 0 | 0 | 0 | 0 | 0 | 0 | 0 | 0 | 0 | 0 | 0 | 0 |
| *Alauda gulgula* | 0 | 0 | 0 | 0 | 0 | 0 | 0 | 0 | 0 | 0 | 0 | 0 | 0 | 0 | 0 | 0 | 0 | 0 | 0 | 0 | 0 | 0 | 0 | 2 | 0 | 0 | 0 | 0 |
| *Alophoixus pallidus* | 0 | 0 | 0 | 0 | 0 | 0 | 0 | 0 | 0 | 0 | 1 | 0 | 0 | 0 | 0 | 0 | 0 | 0 | 0 | 0 | 0 | 0 | 0 | 0 | 0 | 0 | 0 | 0 |
| *Carduelis sinica* | 0 | 0 | 0 | 0 | 0 | 0 | 0 | 0 | 0 | 0 | 0 | 0 | 0 | 0 | 0 | 0 | 0 | 0 | 0 | 0 | 0 | 0 | 0 | 0 | 35 | 0 | 0 | 0 |
| *Centropus bengalensis* | 0 | 0 | 0 | 0 | 0 | 0 | 0 | 0 | 0 | 0 | 0 | 0 | 0 | 0 | 0 | 0 | 0 | 0 | 0 | 0 | 0 | 0 | 0 | 0 | 1 | 0 | 0 | 0 |
| *Corvus splendens* | 0 | 0 | 0 | 0 | 0 | 0 | 0 | 0 | 0 | 0 | 0 | 0 | 0 | 0 | 0 | 0 | 0 | 0 | 0 | 0 | 0 | 0 | 0 | 0 | 0 | 6 | 0 | 0 |
| *Dicrurus hottentottus* | 0 | 0 | 0 | 0 | 0 | 0 | 0 | 0 | 0 | 0 | 0 | 0 | 0 | 0 | 1 | 0 | 0 | 0 | 0 | 0 | 0 | 0 | 0 | 0 | 0 | 0 | 0 | 0 |
| *Pterorhinus sannio* | 0 | 0 | 0 | 0 | 0 | 0 | 0 | 0 | 0 | 0 | 0 | 0 | 0 | 0 | 0 | 0 | 0 | 0 | 0 | 0 | 0 | 0 | 3 | 0 | 0 | 0 | 0 | 0 |
| *Hierococcyx sparverioides* | 0 | 0 | 0 | 0 | 0 | 0 | 0 | 0 | 0 | 0 | 0 | 0 | 0 | 0 | 0 | 0 | 0 | 1 | 0 | 0 | 0 | 0 | 0 | 0 | 0 | 0 | 0 | 0 |
| *Hodgsonius phoenicuroides* | 0 | 0 | 0 | 0 | 0 | 0 | 0 | 0 | 0 | 0 | 0 | 1 | 0 | 0 | 0 | 0 | 0 | 0 | 0 | 0 | 0 | 0 | 0 | 0 | 0 | 0 | 0 | 0 |
| *Lonchura striata* | 0 | 0 | 0 | 0 | 0 | 0 | 0 | 0 | 0 | 0 | 0 | 0 | 0 | 0 | 0 | 0 | 0 | 10 | 0 | 0 | 0 | 0 | 0 | 0 | 0 | 0 | 0 | 0 |
| *Lophura nycthemera* | 0 | 2 | 0 | 0 | 0 | 0 | 0 | 0 | 0 | 0 | 0 | 0 | 0 | 0 | 0 | 0 | 0 | 0 | 0 | 0 | 0 | 0 | 0 | 0 | 0 | 0 | 0 | 0 |
| *Monticola rufiventris* | 0 | 0 | 0 | 0 | 0 | 0 | 0 | 0 | 0 | 0 | 0 | 0 | 0 | 0 | 0 | 0 | 0 | 0 | 0 | 0 | 0 | 0 | 2 | 0 | 0 | 0 | 0 | 0 |
| *Pericrocotus brevirostris* | 0 | 0 | 0 | 0 | 0 | 0 | 0 | 0 | 0 | 0 | 0 | 0 | 0 | 0 | 0 | 0 | 0 | 0 | 0 | 0 | 4 | 0 | 0 | 0 | 0 | 0 | 0 | 0 |
| *Pericrocotus flammeus* | 0 | 0 | 0 | 0 | 0 | 0 | 0 | 0 | 0 | 0 | 5 | 0 | 0 | 0 | 0 | 0 | 0 | 0 | 0 | 0 | 0 | 0 | 0 | 0 | 0 | 0 | 0 | 0 |
| *Pitta oatesi* | 0 | 0 | 0 | 0 | 2 | 0 | 0 | 0 | 0 | 0 | 0 | 0 | 0 | 0 | 0 | 0 | 0 | 0 | 0 | 0 | 0 | 0 | 0 | 0 | 0 | 0 | 0 | 0 |
| *Pteruthius melanotis* | 0 | 0 | 0 | 0 | 0 | 0 | 0 | 0 | 1 | 0 | 0 | 0 | 0 | 0 | 0 | 0 | 0 | 0 | 0 | 0 | 0 | 0 | 0 | 0 | 0 | 0 | 0 | 0 |
| *Tyto capensis* | 0 | 0 | 2 | 0 | 0 | 0 | 0 | 0 | 0 | 0 | 0 | 0 | 0 | 0 | 0 | 0 | 0 | 0 | 0 | 0 | 0 | 0 | 0 | 0 | 0 | 0 | 0 | 0 |
| *Yuhina castaniceps* | 0 | 0 | 0 | 0 | 0 | 0 | 0 | 0 | 0 | 0 | 13 | 0 | 0 | 0 | 0 | 0 | 0 | 0 | 0 | 0 | 0 | 0 | 0 | 0 | 0 | 0 | 0 | 0 |
| *Arachnothera magna* | 0 | 0 | 0 | 0 | 0 | 2 | 0 | 0 | 0 | 0 | 1 | 0 | 0 | 0 | 0 | 0 | 0 | 0 | 0 | 0 | 0 | 0 | 0 | 0 | 0 | 0 | 0 | 0 |
| *Cettia fortipes* | 0 | 0 | 0 | 0 | 0 | 0 | 0 | 0 | 0 | 0 | 0 | 3 | 0 | 0 | 0 | 0 | 0 | 0 | 0 | 0 | 0 | 0 | 0 | 0 | 0 | 4 | 0 | 0 |
| *Dicaeum ignipectus* | 0 | 0 | 0 | 0 | 0 | 0 | 2 | 0 | 0 | 0 | 0 | 0 | 0 | 0 | 0 | 0 | 0 | 0 | 0 | 0 | 0 | 0 | 0 | 0 | 0 | 0 | 2 | 0 |
| *Eumyias thalassina* | 0 | 0 | 0 | 0 | 0 | 3 | 0 | 0 | 0 | 0 | 0 | 0 | 0 | 0 | 0 | 0 | 0 | 1 | 0 | 0 | 0 | 0 | 0 | 0 | 0 | 0 | 0 | 0 |
| *Gallus gallus* | 0 | 0 | 0 | 0 | 0 | 0 | 0 | 0 | 0 | 0 | 1 | 0 | 0 | 0 | 0 | 0 | 0 | 0 | 2 | 0 | 0 | 0 | 0 | 0 | 0 | 0 | 0 | 0 |
| *Leiothrix argentauris* | 0 | 0 | 0 | 0 | 0 | 0 | 0 | 0 | 0 | 0 | 0 | 20 | 0 | 0 | 0 | 0 | 0 | 0 | 0 | 0 | 0 | 0 | 0 | 0 | 0 | 0 | 0 | 40 |
| *Niltava davidi* | 0 | 0 | 0 | 0 | 0 | 0 | 0 | 0 | 0 | 0 | 0 | 0 | 0 | 1 | 0 | 0 | 1 | 0 | 0 | 0 | 0 | 0 | 0 | 0 | 0 | 0 | 0 | 0 |
| *Pycnonotus aurigaster* | 0 | 0 | 0 | 0 | 0 | 0 | 0 | 0 | 0 | 0 | 0 | 0 | 0 | 0 | 0 | 0 | 0 | 0 | 0 | 0 | 0 | 0 | 9 | 1 | 0 | 0 | 0 | 0 |
| *Pycnonotus flavescens* | 0 | 0 | 0 | 0 | 0 | 0 | 0 | 0 | 0 | 0 | 0 | 3 | 0 | 0 | 0 | 0 | 0 | 0 | 0 | 0 | 0 | 0 | 0 | 0 | 2 | 0 | 0 | 0 |
| *Streptopelia tranquebarica* | 0 | 0 | 0 | 0 | 0 | 0 | 0 | 0 | 0 | 0 | 0 | 0 | 0 | 0 | 0 | 0 | 0 | 2 | 0 | 0 | 0 | 0 | 0 | 2 | 0 | 0 | 0 | 0 |
| *Urocissa erythrorhyncha* | 0 | 0 | 0 | 0 | 0 | 0 | 0 | 0 | 0 | 0 | 0 | 0 | 0 | 1 | 0 | 0 | 0 | 0 | 0 | 0 | 0 | 1 | 0 | 0 | 0 | 0 | 0 | 0 |
| *Anthus hodgsoni* | 0 | 0 | 0 | 0 | 0 | 0 | 0 | 0 | 0 | 0 | 0 | 0 | 0 | 0 | 0 | 0 | 0 | 0 | 0 | 0 | 0 | 0 | 0 | 6 | 0 | 0 | 5 | 10 |
| *Hypsipetes mcclellandii* | 0 | 0 | 0 | 2 | 0 | 0 | 0 | 3 | 0 | 0 | 0 | 0 | 0 | 0 | 0 | 0 | 0 | 0 | 0 | 0 | 0 | 0 | 0 | 0 | 0 | 0 | 0 | 0 |
| *Oriolus traillii* | 2 | 0 | 0 | 0 | 0 | 0 | 0 | 0 | 0 | 0 | 2 | 0 | 6 | 0 | 0 | 0 | 0 | 0 | 0 | 0 | 0 | 0 | 0 | 0 | 0 | 0 | 0 | 0 |
| *Pnoepyga pusilla* | 0 | 0 | 0 | 0 | 0 | 0 | 0 | 2 | 0 | 0 | 0 | 0 | 0 | 0 | 0 | 0 | 0 | 0 | 0 | 2 | 0 | 0 | 0 | 0 | 0 | 0 | 0 | 2 |
| *Saxicola ferrea* | 0 | 0 | 0 | 0 | 0 | 0 | 0 | 0 | 0 | 0 | 0 | 0 | 0 | 0 | 0 | 0 | 0 | 0 | 0 | 0 | 0 | 0 | 0 | 10 | 6 | 0 | 3 | 0 |
| *Abroscopus superciliaris* | 0 | 0 | 0 | 0 | 3 | 0 | 0 | 0 | 0 | 0 | 0 | 0 | 0 | 1 | 0 | 0 | 1 | 0 | 0 | 0 | 0 | 0 | 0 | 0 | 0 | 0 | 0 | 0 |
| *Chalcophaps indica* | 0 | 0 | 0 | 0 | 1 | 0 | 4 | 0 | 1 | 1 | 0 | 0 | 0 | 0 | 0 | 0 | 0 | 0 | 0 | 0 | 0 | 0 | 0 | 0 | 0 | 0 | 0 | 0 |
| *Enicurus leschenaulti* | 0 | 0 | 0 | 0 | 1 | 0 | 0 | 0 | 3 | 0 | 0 | 0 | 0 | 0 | 0 | 0 | 1 | 0 | 0 | 0 | 0 | 0 | 0 | 0 | 0 | 0 | 0 | 0 |
| *Lanius schach* | 0 | 0 | 0 | 0 | 0 | 0 | 0 | 0 | 0 | 0 | 1 | 0 | 0 | 0 | 0 | 0 | 0 | 4 | 0 | 0 | 0 | 0 | 0 | 5 | 0 | 0 | 3 | 0 |
| *Megalaima virens* | 0 | 1 | 0 | 0 | 0 | 0 | 0 | 0 | 0 | 1 | 0 | 0 | 0 | 0 | 2 | 0 | 0 | 0 | 0 | 0 | 0 | 0 | 0 | 0 | 0 | 1 | 0 | 0 |
| *Phaenicophaeus tristis* | 0 | 0 | 0 | 0 | 0 | 0 | 0 | 0 | 1 | 0 | 0 | 0 | 0 | 2 | 0 | 0 | 0 | 0 | 1 | 0 | 1 | 0 | 0 | 0 | 0 | 0 | 0 | 0 |
| *Psarisomus dalhousiae* | 0 | 2 | 0 | 1 | 0 | 0 | 0 | 0 | 0 | 0 | 0 | 0 | 1 | 0 | 1 | 0 | 0 | 0 | 0 | 0 | 0 | 0 | 0 | 0 | 0 | 0 | 0 | 0 |
| *Serilophus lunatus* | 1 | 0 | 0 | 0 | 0 | 2 | 0 | 0 | 0 | 0 | 2 | 0 | 0 | 0 | 0 | 0 | 0 | 0 | 0 | 0 | 0 | 0 | 0 | 0 | 0 | 0 | 1 | 0 |
| *Sitta nagaensis* | 0 | 0 | 0 | 0 | 0 | 1 | 0 | 0 | 0 | 2 | 0 | 1 | 0 | 0 | 0 | 0 | 0 | 0 | 0 | 0 | 0 | 0 | 0 | 0 | 0 | 0 | 0 | 1 |
| *Alcippe dubia* | 0 | 0 | 0 | 0 | 0 | 0 | 0 | 0 | 0 | 2 | 0 | 0 | 0 | 2 | 0 | 0 | 0 | 0 | 0 | 0 | 0 | 2 | 5 | 0 | 0 | 0 | 0 | 2 |
| *Bambusicola fytchii* | 0 | 0 | 0 | 0 | 0 | 0 | 0 | 0 | 0 | 0 | 0 | 1 | 0 | 0 | 0 | 0 | 0 | 0 | 0 | 0 | 0 | 0 | 9 | 0 | 1 | 2 | 1 | 0 |
| *Macronous gularis* | 0 | 0 | 0 | 0 | 0 | 0 | 0 | 0 | 0 | 0 | 2 | 0 | 1 | 4 | 0 | 0 | 0 | 0 | 0 | 0 | 0 | 0 | 0 | 0 | 4 | 0 | 0 | 1 |
| *Parus major* | 0 | 0 | 0 | 0 | 0 | 0 | 0 | 0 | 0 | 0 | 0 | 1 | 0 | 0 | 0 | 0 | 0 | 0 | 0 | 0 | 0 | 0 | 2 | 0 | 4 | 0 | 3 | 1 |
| *Prinia hodgsonii* | 0 | 0 | 0 | 0 | 0 | 0 | 0 | 0 | 0 | 0 | 7 | 0 | 0 | 0 | 0 | 0 | 0 | 0 | 0 | 0 | 0 | 0 | 14 | 35 | 14 | 0 | 10 | 0 |
| *Yuhina flavicollis* | 0 | 0 | 0 | 15 | 0 | 0 | 0 | 15 | 0 | 0 | 0 | 0 | 45 | 0 | 15 | 35 | 0 | 0 | 0 | 0 | 0 | 0 | 0 | 0 | 0 | 0 | 0 | 0 |
| *Yuhina zantholeuca* | 0 | 0 | 0 | 0 | 0 | 0 | 0 | 3 | 0 | 0 | 0 | 0 | 1 | 1 | 0 | 0 | 0 | 0 | 0 | 1 | 0 | 0 | 0 | 0 | 1 | 0 | 0 | 0 |
| *Arborophila rufogularis* | 1 | 1 | 0 | 0 | 3 | 0 | 0 | 0 | 0 | 0 | 0 | 1 | 0 | 0 | 3 | 0 | 0 | 0 | 0 | 0 | 0 | 0 | 0 | 0 | 0 | 1 | 0 | 0 |
| *Brachypteryx montana* | 0 | 0 | 0 | 0 | 4 | 0 | 0 | 8 | 0 | 0 | 0 | 2 | 0 | 1 | 0 | 0 | 0 | 0 | 0 | 8 | 1 | 0 | 0 | 0 | 0 | 0 | 0 | 0 |
| *Myophonus caeruleus* | 1 | 0 | 0 | 0 | 1 | 0 | 0 | 0 | 1 | 0 | 2 | 0 | 0 | 0 | 0 | 4 | 0 | 0 | 0 | 1 | 0 | 0 | 0 | 0 | 0 | 0 | 0 | 0 |
| *Napothera epilepidota* | 0 | 1 | 0 | 0 | 0 | 5 | 1 | 0 | 0 | 0 | 0 | 0 | 0 | 0 | 1 | 0 | 1 | 0 | 0 | 0 | 0 | 0 | 0 | 0 | 0 | 0 | 0 | 0 |
| *Ninox scutulata* | 0 | 0 | 0 | 0 | 0 | 0 | 0 | 0 | 0 | 0 | 1 | 0 | 1 | 1 | 0 | 0 | 0 | 0 | 0 | 1 | 0 | 0 | 0 | 0 | 3 | 0 | 0 | 1 |
| *Prinia flaviventris* | 6 | 0 | 0 | 0 | 0 | 0 | 0 | 0 | 0 | 0 | 8 | 0 | 0 | 0 | 0 | 0 | 0 | 20 | 0 | 0 | 0 | 0 | 64 | 0 | 0 | 9 | 0 | 6 |
| *Pycnonotus jocosus* | 0 | 0 | 0 | 0 | 0 | 0 | 0 | 0 | 0 | 0 | 1 | 0 | 0 | 0 | 0 | 0 | 0 | 0 | 0 | 0 | 5 | 0 | 5 | 1 | 0 | 0 | 6 | 2 |
| *Tesia castaneocoronata* | 0 | 0 | 0 | 0 | 1 | 0 | 0 | 0 | 3 | 1 | 0 | 0 | 1 | 0 | 0 | 0 | 0 | 1 | 0 | 0 | 0 | 0 | 0 | 0 | 0 | 0 | 0 | 0 |
| *Treron sphenura* | 1 | 0 | 1 | 0 | 0 | 1 | 0 | 0 | 0 | 0 | 0 | 0 | 0 | 0 | 2 | 0 | 0 | 0 | 2 | 0 | 0 | 0 | 0 | 0 | 1 | 0 | 0 | 0 |
| *Phylloscopus davisoni* | 0 | 0 | 0 | 0 | 0 | 0 | 0 | 0 | 0 | 0 | 3 | 2 | 3 | 1 | 0 | 0 | 0 | 0 | 0 | 0 | 0 | 0 | 1 | 0 | 3 | 0 | 0 | 6 |
| *Pomatorhinus hypoleucos* | 0 | 0 | 0 | 0 | 0 | 0 | 0 | 0 | 0 | 0 | 3 | 0 | 0 | 0 | 0 | 0 | 0 | 0 | 0 | 0 | 1 | 0 | 9 | 0 | 1 | 19 | 5 | 1 |
| *Tesia olivea* | 0 | 0 | 0 | 0 | 7 | 4 | 0 | 0 | 2 | 7 | 0 | 0 | 0 | 0 | 0 | 0 | 1 | 0 | 0 | 1 | 0 | 0 | 0 | 0 | 0 | 0 | 0 | 0 |
| *Pycnonotus xanthorrhous* | 0 | 0 | 0 | 0 | 0 | 0 | 0 | 0 | 0 | 0 | 1 | 3 | 0 | 0 | 0 | 0 | 0 | 0 | 0 | 0 | 1 | 3 | 17 | 0 | 1 | 15 | 56 | 5 |
| *Chloropsis hardwickii* | 0 | 0 | 0 | 0 | 5 | 0 | 0 | 2 | 0 | 0 | 2 | 4 | 2 | 0 | 0 | 0 | 2 | 0 | 0 | 6 | 0 | 0 | 2 | 0 | 0 | 0 | 0 | 2 |
| *Prinia superciliaris* | 0 | 0 | 0 | 0 | 0 | 0 | 0 | 0 | 0 | 2 | 2 | 7 | 0 | 0 | 0 | 0 | 0 | 9 | 0 | 0 | 1 | 0 | 11 | 5 | 5 | 11 | 19 | 8 |
| *Cyornis banyumas* | 0 | 0 | 5 | 0 | 0 | 3 | 0 | 0 | 0 | 0 | 2 | 1 | 2 | 0 | 5 | 0 | 1 | 0 | 1 | 0 | 8 | 0 | 0 | 0 | 3 | 2 | 0 | 0 |
| *Glaucidium cuculoides* | 0 | 5 | 0 | 2 | 2 | 2 | 2 | 0 | 0 | 0 | 0 | 0 | 0 | 0 | 2 | 1 | 0 | 2 | 1 | 0 | 0 | 1 | 0 | 2 | 0 | 0 | 0 | 0 |
| *Hypsipetes leucocephalus* | 0 | 0 | 51 | 0 | 3 | 0 | 0 | 0 | 0 | 0 | 0 | 2 | 0 | 1 | 0 | 0 | 10 | 0 | 5 | 1 | 3 | 0 | 0 | 0 | 2 | 0 | 0 | 24 |
| *Minla cyanouroptera* | 0 | 0 | 0 | 6 | 27 | 0 | 0 | 6 | 6 | 24 | 0 | 0 | 0 | 0 | 0 | 0 | 24 | 20 | 27 | 11 | 50 | 0 | 0 | 0 | 0 | 0 | 0 | 0 |
| *Pomatorhinus ruficollis* | 0 | 0 | 4 | 1 | 3 | 4 | 0 | 0 | 3 | 0 | 1 | 6 | 2 | 0 | 0 | 0 | 3 | 0 | 1 | 6 | 2 | 0 | 0 | 0 | 0 | 0 | 0 | 0 |
| *Hemixos flavala* | 18 | 8 | 1 | 0 | 0 | 0 | 0 | 3 | 0 | 0 | 14 | 0 | 12 | 1 | 7 | 3 | 0 | 0 | 1 | 0 | 0 | 0 | 0 | 0 | 4 | 0 | 0 | 6 |
| *Rhipidura albicollis* | 0 | 0 | 0 | 0 | 4 | 0 | 1 | 0 | 1 | 1 | 3 | 0 | 1 | 4 | 0 | 4 | 4 | 4 | 0 | 5 | 1 | 0 | 0 | 0 | 0 | 0 | 0 | 0 |
| *Culicicapa ceylonensis* | 2 | 0 | 0 | 2 | 3 | 4 | 13 | 3 | 0 | 3 | 0 | 0 | 3 | 9 | 1 | 8 | 4 | 2 | 0 | 0 | 0 | 0 | 0 | 0 | 2 | 0 | 0 | 0 |
| *Pycnonotus melanicterus* | 2 | 0 | 8 | 0 | 0 | 0 | 1 | 5 | 1 | 0 | 18 | 2 | 12 | 1 | 6 | 2 | 2 | 7 | 3 | 0 | 4 | 0 | 0 | 0 | 2 | 2 | 0 | 14 |
| *Stachyris chrysaea* | 0 | 0 | 2 | 6 | 3 | 5 | 0 | 0 | 1 | 1 | 2 | 1 | 1 | 0 | 0 | 0 | 4 | 0 | 1 | 2 | 1 | 1 | 0 | 0 | 1 | 1 | 0 | 1 |
| *Aethopyga gouldiae* | 0 | 5 | 2 | 0 | 1 | 2 | 5 | 3 | 0 | 2 | 5 | 6 | 8 | 3 | 11 | 3 | 2 | 0 | 7 | 5 | 4 | 1 | 0 | 0 | 0 | 4 | 0 | 0 |
| *Zosterops Spp.* | 20 | 0 | 0 | 20 | 0 | 20 | 0 | 20 | 0 | 0 | 70 | 220 | 36 | 20 | 16 | 20 | 60 | 80 | 48 | 50 | 80 | 60 | 0 | 10 | 40 | 0 | 0 | 160 |
| *Dicaeum concolor* | 0 | 0 | 6 | 2 | 1 | 1 | 0 | 16 | 6 | 4 | 7 | 7 | 1 | 1 | 4 | 0 | 6 | 10 | 7 | 3 | 13 | 6 | 0 | 0 | 0 | 0 | 1 | 9 |
| *Dicrurus macrocercus* | 1 | 2 | 0 | 2 | 2 | 2 | 0 | 1 | 0 | 0 | 4 | 7 | 7 | 6 | 1 | 1 | 2 | 0 | 3 | 4 | 0 | 0 | 3 | 0 | 4 | 1 | 3 | 1 |
| *Alcippe morrisonia* | 22 | 11 | 33 | 80 | 105 | 73 | 11 | 105 | 65 | 126 | 0 | 30 | 83 | 15 | 45 | 61 | 100 | 0 | 85 | 95 | 188 | 33 | 0 | 0 | 0 | 0 | 0 | 78 |
| *Orthotomus cuculatus* | 3 | 0 | 0 | 9 | 5 | 19 | 9 | 5 | 7 | 9 | 0 | 2 | 1 | 0 | 1 | 3 | 6 | 1 | 6 | 4 | 3 | 2 | 0 | 1 | 2 | 0 | 0 | 2 |
| *Psilopogon asiatica* | 2 | 10 | 4 | 12 | 10 | 1 | 9 | 8 | 2 | 14 | 4 | 0 | 10 | 0 | 3 | 13 | 7 | 1 | 8 | 8 | 3 | 4 | 0 | 3 | 10 | 5 | 8 | 9 |
